# In Vitro Antimicrobial Activity of Medicinal Plant Extracts against Some Bacterial Pathogens Isolated from Raw and Processed Meat

**DOI:** 10.3390/life11111178

**Published:** 2021-11-04

**Authors:** Ahmed Kh. Meshaal, Helal F. Hetta, Ramadan Yahia, Khamael M. Abualnaja, Abdallah Tageldein Mansour, Israa M. S. Al-Kadmy, Saad Alghamdi, Anas S. Dablool, Talha Bin Emran, Haitham Sedky, Gaber El-Saber Batiha, Waleed El-Kazzaz

**Affiliations:** 1 Department of Botany and Microbiology, Faculty of Science, Al-Azhar University, Assiut 71524, Egypt; ahmad_meshaal1650@yahoo.com; 2 Department of Medical Microbiology and Immunology, Faculty of Medicine, Assuit University, Assuit 71515, Egypt; 3 Department of Microbiology and Immunology, Faculty of Pharmacy, Deraya University, Minia 61512, Egypt; ramadanfarrag@rocketmail.com; 4 Department of Chemistry, College of Science, Taif University, Taif 21944, Saudi Arabia; k.ala@tu.edu.sa; 5 Animal and Fish Production Department, College of Agricultural and Food Sciences, King Faisal University, Al-Ahsa 31982, Saudi Arabia; amansour@kfu.edu.sa; 6 Fish and Animal Production Department, Faculty of Agriculture (Saba Basha), Alexandria University, Alexandria 21531, Egypt; 7 Faculty of Science and Engineering, School of Engineering, University of Plymouth, Plymouth PL4 8AA, UK; israa.al-kadmy@plymouth.ac.uk; 8 Branch of Biotechnology, Department of Biology, College of Science, Mustansiriyah University, Baghdad 10244, Iraq; 9 Laboratory Medicine Department, Faculty of Applied Medical Sciences, Umm Al-Qura University, Makkah 24381, Saudi Arabia; ssalghamdi@uqu.edu.sa; 10 Department of Public Health, Health Sciences College at Al-Leith, Umm Al-Qura University, Makkah 24381, Saudi Arabia; Asdablool@uqu.edu.sa; 11 Department of Pharmacy, BGC Trust University Bangladesh, Chittagong 4381, Bangladesh; talhabmb@bgctub.ac.bd; 12 Department of Microbiology and Immunology, Faculty of Pharmacy, Assiut University, Assiut 71515, Egypt; haitham.mohamed@aun.edu.eg; 13 Department of Pharmacology and Therapeutics, Faculty of Veterinary Medicine, Damanhour University, Damanhur 22511, Egypt; dr_gaber_batiha@vetmed.dmu.edu.eg; 14 Molecular Microbiology Lab., Botany Department, Faculty of Science, Suez Canal University, Ismailia 41522, Egypt; walid_elkazaz@science.suez.edu.eg

**Keywords:** *Staphylococcus aureus*, *E. coli O157: H7*, *Klebsiella pneumonia*, 16S rRNA, antimicrobial, in vitro, spectrum, antibacterial and preservatives

## Abstract

Background and aim: The poultry meat and its products are considered ideal media for bacterial growth and spoilage, as they are highly nutritive with a favorable pH. The food industry has focused its attention on a great diversity of plant species as food preservatives. The aim of this study was to investigate the presence of *Staphylococcus aureus*, *Escherichia coli* *O157: H7*, and *Klebsiella pneumonia* in food samples and to evaluate of the antibacterial activity of some medicinal plant extracts against these bacteria. Methods: Raw and processed meat samples (*n* = 60) were collected from abattoirs and local markets. *S. aureus, E. coli O157: H7,* and *K. pneumonia* were isolated, identified by phenotypic methods, and then confirmed by 16S rRNA gene sequencing. The antibacterial activity and spectrum of essential oils and spices powder of cumin, black seeds, cloves, cinnamon, and marjoram was determined against the isolated strains in this study by microbial count and well-diffusion techniques. Results: A total of 33 isolates have been identified as *S. aureus*, 30 isolates were identified as *E. coli O157: H7*, and 15 isolates were identified as *K. pneumonia*. *S. aureus, E. coli O157: H7*, and *K. pneumonia* could be detected in both fresh and processed food with higher prevalence in the processed meat. There was a significant decrease in microbial count in treated samples either with the spices powder or essential oils of the tested medicinal plants compared to control samples during storage time period. Furthermore, while the microbial count increased in the control samples, the microbial count decreased to reach zero in almost all treated samples with essential oils after 15 days of storage. Conclusion: *S. aureus, E. coli O157: H7*, and *K. pneumonia* are associated with food from animal sources, in either fresh or processed meat samples. The prevalence of them was higher in the processed meat than in fresh meat. The essential oils and spices powder of cumin, black seeds, cloves, cinnamon, and marjoram have an in vitro wide spectrum antibacterial activity with the highest antibacterial activity for the black seeds.

## 1. Introduction

The food industry has focused attention on a great diversity of plant species as food preservatives. Such plants are composed of bioactive compounds that shield plants from microbiological attacks, but have also be manipulated and used by humans for thousands of years as food and medicinal sources [1]. Essential oils and their components are gaining increasing interest because of their wide acceptance by consumers, and their exploitation for potential multi-purpose functional use [2].

Because of their preservative properties, plants have a high potential as natural food additives based on their wide spectrum of bioactive compounds [3]. One of the potential methods to tackle the twin concerns of food security and environmental sustainability is to use beneficial phytomicrobiome (i.e., bacteria closely connected with plant tissues). A variety of vital bacteria can be found in numerous areas of the plant, including the root, shoot, leaf, seed, and flower, all of which play important roles in plant health, development, and productivity, and could directly help to improve the quality and amount of food produced. Increased resource utilization efficiency and resilience to biotic and abiotic challenges are also benefits of the phytomicrobiome [4].

Regrettably, the poultry meat and its products are an ideal medium for bacterial growth and spoilage, as they are highly nutritive with a favorable pH [5]. *Staphylococcus aureus* (*S. aureus*) is a spherical Gram-positive bacterium which is capable of producing a highly heat-stable protein toxin and causing gastroenteritis and food poisoning in humans [6]. Methicillin-resistant Staphylococcus aureus (MRSA) can cause infections in human and animals, and it is now considered as one of the most significant zoonotic infections [7,8]. MRSA has been isolated from several non-human sources, such as bovine milk, pets, and chicken meats [9,10,11], suggesting the potential of human infection with MRSA from food [12,13].

*Escherichia coli* (*E. coli*) and *Klebsiella pneumonia (K. pneumonia)* are members of Enterobacteriaceae, and can cause a number of nosocomial infections and may be acquired from the environment (foodborne) [14,15,16,17]. Although it is regarded as part of the flora of the human intestinal tract, several highly adapted *E. coli* clones have evolved and developed the ability to cause disease in several areas of the human body [18,19,20]. *Klebsiella species* are opportunistic bacteria that are commonly found in a wide range of animals, in the environment, and in the gastrointestinal tracts of animals especially those raised for human consumption [15]. *K. pneumonia* can contaminate meat and dairy products and lead to illness and food spoilage [21,22,23]. Such bacteria are common sources of food contamination by feces (of both animal and human origin), personnel, water, and containers [24].

Generally, the control of bacterial pathogens depends on antimicrobial therapy, but the development of bacterial resistance has led to the search for new options. The use of essential oils in this regard represents a promising alternative [25]. Persister cells and viable but non-culturable (VBNC) cells could survive during exposure to high doses of antibiotics. Persister cells survive and defy the antibiotic, and regrow again on culture media after removing the antibiotic, whilst VBNC cells survive and defy the antibiotic but may resume growth only after a long and specified treatment. Thus, it is difficult to study and detect VBNC cells using standard microbiological experiments. VBNC cells establish a prime public health concern, thus, they have been reported in 51 human pathogens and they are hard to be eradicated through standard sterilization actions, such as heat, acid, ethanol, or even by antibiotic or osmotic stress [26,27,28,29,30,31,32,33]. In this sense, the use of essential oils as food preservatives constitute a promising alternative [25].

The essential oils are secondary metabolites that can be extracted from different parts of plants. Essential oils can exert antioxidant and antimicrobial effects by reducing the population of pathogenic bacteria. Essential oils can prevent food deterioration and degradation by inhibiting the growth of foodborne pathogens and acting as antioxidants [34].

The aim of this study was to evaluate the antibacterial activity of essential oils and spices powder of cumin, black seeds, cloves, cinnamon, and marjoram against *S. aureus*, *E. coli O157: H7*, and *K. pneumonia* isolated from raw and processed meat samples.

## 2. Materials and Methods

### 2.1. Collection of Meat Samples

To purify new isolates from *E. coli O157: H7*, *S. aureus*, and *K. pneumonia*, 60 meat samples including 15 raw meat samples (beef, mutton, and chicken) and 45 processed meat samples (ground beef, beef burgers, beef sausage, ground chicken, and chicken burgers) were collected throughout three seasons, the first between 21 June to 22 September (summer 2019), the second between 23 September to 20 December (autumn 2019), and the last season between 21 December to 20 March (winter 2020). Samples were collected from abattoirs and markets located in Assiut city, Egypt. The samples were cut and minced just before analysis and treatments.

### 2.2. Isolation and Identification of Some Bacterial Species in the Meat Samples

Mannitol salt agar medium (Biolife Italiana S.r.l., Viale Monza 272, 20128 Milan, Italy) was used for isolation and purification of *S. aureus* [35]. Sorbitol MacConkey agar (SMA) (Difco) and Eosin methylene blue (EMB) agar (Biolife Italiana S.r.l., Viale Monza 272, 20128 Milan, Italy) were used for isolation and purification of *E. coli O157: H7* [36]. MacConkey agar was used for isolation and purification of *K. pneumonia*. Biochemical identification was done on pure colony from each strain with specific criteria on selective culture media, as described previously [37,38].

DNA was extracted from a single pure colony from each isolate using QIAamp DNA Mini Kit (Qiagen, Germantown, MD, USA). The 16S rRNA was amplified using the universal primers 27F and 1492R, as previously described by Lane [39]. The PCR conditions were as follows: 94 °C for 5 min followed by 30 cycles of 40 s at 94 °C, 30 s at 52 °C, and 1 min at 72 °C. A final 7 min extension step was done at 72 °C [40,41].

The PCR amplicons were purified using the QIAquick gel extraction kit (Qiagen, Germantown, MD, USA) then sequenced using the Applied Biosystem Automated 3730XL DNA sequencer (Solgent Company, Daejeon, South Korea). The obtained sequences were further analyzed using BLAST tool from the National Center of Biotechnology Information (NCBI) website. Phylogenetic analysis of the sequence was conducted using the software MEGA 6.0 [42].

### 2.3. Evaluation of the Antibacterial Activities of the Medicinal Plants against the Bacteria Isolated from Meat Samples by Well-Diffusion Technique

Antibacterial activities of essential oils and spices powder were determined by the agar well-diffusion as previously described [41,43,44,45,46]. Briefly, nutrient agar plates were inoculated with 0.2 mL of a freshly prepared 24 h culture (10^8^ CFU/mL) of the isolated *S. aureus, E. coli O157: H7* and *K. pneumonia*. A sterile cork borer was used to make a 6 mm well in the agar. Serial dilutions from each tested essential oil were made (% *v*/*v*) in dimethyl sulfoxide (DMSO) (10% aqueous) solvent as 50 and 100 mg/mL. The concentrations made from each spices powder were 50 and 100 mg/mL in ethyl alcohol [47]. Each well in the inoculated agar plates was filled with 50 μL of each tested essential or spices powder preparation, then the plates were incubated for 24 h at 35 °C and the inhibition zones were measured [44,45,46].

### 2.4. Assessment of the Impact of Essential Oil and Spices Powder on the Microbial Count in Tested Meat Samples

Different meat product samples were sliced into small pieces with a sterile knife. Each meat product sample (in 25 g) was combined with 225 mL of sterile 0.1% peptone solution in a sterile polyethylene bag. To choose the best dilution (bacterial count 30–300 CFU), serial dilutions (10^−1^, 10^−2^, 10^−3^, 10^−4^ and 10^−5^) should be plated in triplicate for bacterial colony counts, thus, dilution number 3 (10^−3^) was chosen [48]. The total bacterial counts were determined by using the plate counts technique on a nutrient or selective agar medium, this was completed by adding 100µ (10^−1^) from the diluted sample as previously described [48]. The calculation was conducted as follows: bacterial count CFU/g = Log_10_ (number of colonies × 10^−1^ × 10^−3^).

The powder samples were collected from the local market in Assiut city, while the plant essential oil samples were collected from the National Research Center, unit of oils extraction, Egypt. Under sterile conditions, minced beef samples were mixed with either spices powder (0.5 and 1% of minced beef weight as weight/weight) or the essential oils (0.25 and 0.5% minced beef weight as volume/weight) of the same plant. Additional groups were kept as a control group. Each sample was packed in polyethylene bags and stored at 4 °C ± 1, and all analysis was conducted at intervals of 0, 3, 6, 9, 12, and 15 days as previously described.

### 2.5. Statistical Analysis

GraphPad Prism program version 8.0.1 (San Diego, CA, USA) was used. *p* values less than 0.05 were considered significant. Kruskal–Wallis test was used to analyze the difference between groups.

## 3. Results

### 3.1. Isolation and Identification of Some Bacterial Species in the Meat Samples

According to the results of culture media and biochemical reactions, 33 (55%) isolates were identified as *S. aureus*. In total, 30 (50%) isolates were identified as *E. coli O157: H7*, and 15 (25%) isolates were identified as *K. pneumonia* among all tested samples (*n* = 60) (Table 1).

The isolates were further confirmed by 16S rRNA gene sequence analysis. The size of the amplified genes was approximately 1500 bp. The basic local alignment search tool (BLAST) at the National Center for Biotechnology Information (NCBI) was used to retrieve homologous sequences in Gen Bank. Azhar1 strain was identified as *S. aureus*, Azhar2 strain was identified as *E. coli O157H7*, and Azhar3 strain was identified as *K. pneumonia*. The three isolates were deposited in the GenBank database at NCBI under the accession numbers MT705745, MT705746, and MT705744, respectively (Table 2).

**Figure 1:**
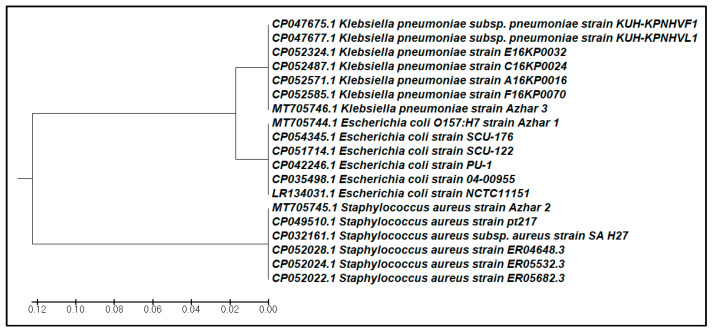
Phylogenetic dendrogram based on 16S rRNA gene sequences showing the position of the selected strains among members of different genus species. The evolutionary history was inferred using the UPGMA method [49]. The tree is drawn to scale, with branch lengths in the same units as those of the evolutionary distances used to infer the phylogenetic tree. The evolutionary distances were computed using the Kimura 2-parameter method [50], and are in the units of the number of base substitutions per site. The analysis involved 19 nucleotide sequences. Codon positions included were the 1st. All positions containing gaps and missing data were eliminated. There was a total of 336 positions in the final dataset. Evolutionary analyses were conducted in MEGA6 [42].

A phylogenetic tree based on the comparison of 16S rRNA gene sequences with reference strains was constructed. The phylogenetic analysis was performed with 15,000 bp and 2000 bp sequences for *S. aureus*, *E. coli*, and *K. pneumonia* using the software MEGA 6 (Figure 1).

### 3.2. Antibacterial Activities of Essential Oils and Spices Powder of the Medicinal Plants against the Isolates by Well-Diffusion Assays Technique

**Figure 2:**
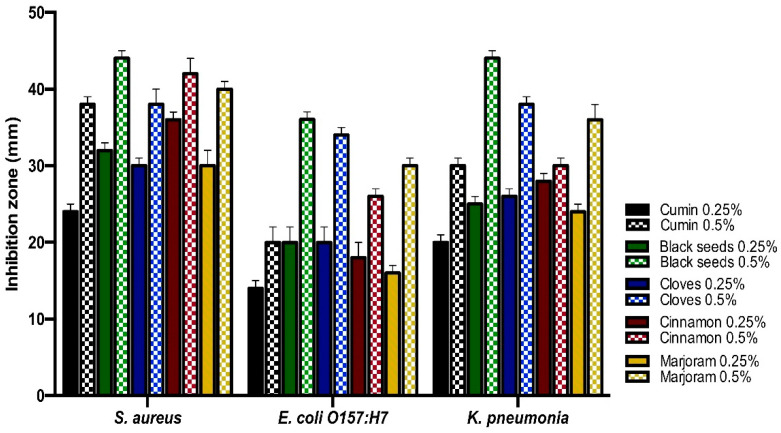
Antibacterial activities of essential oils of the medicinal plants against the isolates by well-diffusion technique.

**Figure 3:**
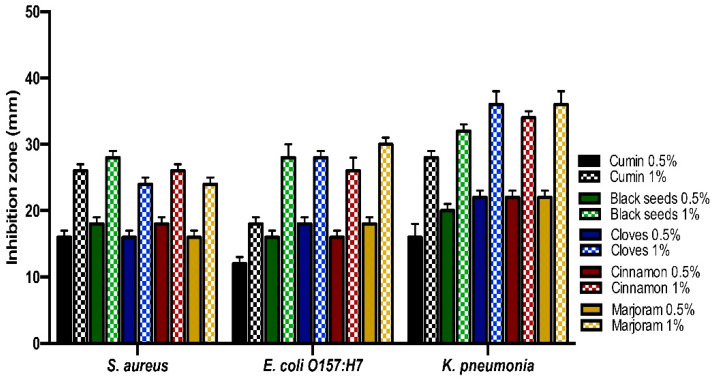
Antibacterial activities of spices powder of the medicinal plants against the isolates by well-diffusion technique.

From the inhibition zone diameter, the antibacterial activity of essential oils was stronger than the spices powder for the same medicinal plant. The black seeds essential oils gave the strongest antibacterial activity against *S. aureus, E. coli O157: H7*, and *K. pneumonia*. The black seeds spices powder gave the strongest antibacterial activity against *S. aureus*, while the marjoram spices powder gave the strongest antibacterial activity against *E. coli O157: H7*. Furthermore, both marjoram and clove spices powder gave the strongest antibacterial activity against *K. pneumonia* (Figure 2 and Figure 3).

### 3.3. Assessment of the Impact of Essential Oil and Spices Powder on the Microbial Count in Tested Meat Samples

The bacterial counts in the control sample and minced beef samples contained either essential oils with concentrations 0.25% and 0.50%, or spices powder with concentrations 0.50% and 1.0%, and were evaluated during storage at 4 ± 1 °C for 15 days with 3-day intervals. After 15 days of storage, there was a statistically significant difference in bacterial count between the control sample and each treated group either with essential oils or spices powder.

#### 3.3.1. Impact of Essential Oil and Spices Powder on the *S. aureus* Bacterial Count in Tested Meat Samples

For essential oils treatment, *S. aureus* counts increased in the control sample from 4.339 log CFU/g at zero time, to 4.464 log CFU/g at the end of storage periods, with mean ± S.D (4.39 ± 0.05). However, *S. aureus* count reached zero in samples containing cumin and black seeds essential oils (0.25% and 0.50%), cloves (0.50%), cinnamon essential oils (0.25% and 0.50%), and marjoram (0.50%) after 12 days of storage. Moreover, after 15 days of storage, the *S. aureus* count reached zero in all treated samples with essential oils either at 0.25 or 0.5%, except the control. Furthermore, the difference in *S. aureus* count between the control group and all the treated samples with essential oils (both at 0.25% and 0.5%) was significant (Table 3).

For spices powder treatment, *S. aureus* counts reached zero after 9 days of storage in minced beef samples treated with the spice powders of black seeds at 0.50% and 1.0% (means ± S.Ds were 2.01 ± 2.2 and 1.98 ± 2.18 log CFU/g, respectively) and cinnamon at level 1.0% (mean ± S.D was 2.01 ± 2.17 log CFU/g). Moreover, at the end of the storage period (15 days), *S. aureus* count reached zero in all samples treated with spices powder (both at 0.5 and 1.0%), except for the control sample in which the count was 4.493 log CFU/g. The difference in *S. aureus* count between the control group and all the treated samples with spices powder (both at 0.5% and 1.0%) was significant (Table 4).

#### 3.3.2. Impact of Essential Oil and Spices Powder on the *E. coli O157: H7* Bacterial Count in Tested Meat Samples

For essential oil treatments, *E. coli O157: H7* count increased in the control sample from 4.125 log CFU/g at zero time, and reached 4.461 log CFU/g at the end of storage periods. The *E. coli O157: H7* count reached zero after 12 days of storage in samples treated with 0.5% black seed oil and clove oil at both 0.25% and 0.5%. Furthermore, after 15 days of the refrigerated storage, the *E. coli O157: H7* count reached zero in all samples treated with essential oils (both at 0.25% and 0.5%), except samples that treated with marjoram oils either at 0.25% or 0.50% and the control sample. The difference in *E. coli O157: H7* count between the control group and all the treated samples with essential oils (both at 0.25% and 0.5%) was significant (Table 5).

For spices powder treatment, *E. coli O157: H7* count increased in the control sample from 4.349 log CFU/g at the start to 4.455 log CFU/g after 15 days of storage with mean ± S.D 4.39 ± 0.039 log CFU/g. The *E. coli O157: H7* count reached zero after 12 days of storage in samples treated with the essential oils of black seeds, clove (both at 0.5% and 1.0%), and 1.0% cinnamon. Furthermore, the *E. coli O157: H7* count reached zero after 15 days of storage in all samples treated with spices powder (both at 0.5% and 1.0%) except for samples that were treated with marjoram at 0.5%. Furthermore, the difference in *E. coli O157: H7* count between the control group and all the treated samples with spices powder (both at 0.5% and 1.0%) was significant (Table 6).

#### 3.3.3. Impact of Essential Oil and Spices Powder on the *K. pneumonia* Bacterial Count in Tested Meat Samples

For essential oil treatments, *K. pneumonia* count increased in the control sample from 3.297 log CFU/g at zero time, and reached 4.479 log CFU/g at the end of storage period. After 12 days of storage, the *K. pneumonia* count reached zero in samples treated with essential oils of cumin, black seeds, and clove at both 0.25% and 0.5% concentrations. In addition, the *K. pneumonia* count reached zero after 12 days of storage for samples treated with 0.5% cinnamon and marjoram oils. However, the *K. pneumonia* count decreased from 4.228 log CFU/g (at zero time) to 3.398 log CFU/g after 12 days of storage for samples treated with 0.25% cinnamon oil. The *K. pneumonia* count decreased from 4.291 log CFU/g at zero time to 3.519 log CFU/g after 12 days of storage for samples treated with 0.25% marjoram oil. Furthermore, the *K. pneumonia* count reached zero after 15 days of storage for all samples treated with both 0.25% and 0.5% essential oils. There was a significant difference in *K. pneumonia* count between the control group and all the treated samples with essential oils (both at 0.25% and 0.5%) (Table 7).

For spices powder treatment, *K. pneumonia* count increased in the control sample from 4.299 log CFU/g at zero time, and reached 4.455 log CFU/g at the end of the storage period. After 12 days of storage, the *K. pneumonia* count reached zero in samples treated with the spices powder of cumin, black seeds, and clove at both 0.5% and 1.0%. In addition, the *K. pneumonia* count decreased from 4.248 log CFU/g at zero time, to reach 3.592 log CFU/g after 12 days of storage for samples treated with 0.50% spices powder of cinnamon, however it reached zero for samples treated with 1.0% spices powder of cinnamon for the same period. The *K. pneumonia* count decreased from 4.297 log CFU/g at zero time, to reach 3.624 log CFU/g after 12 days of storage for samples treated with 0.50% spices powder of marjoram, however it decreased from 4.156 log CFU/g at zero time, to reach 3.042 log CFU/g for samples treated with 1.0% spices powder of marjoram for the same period. Furthermore, the *K. pneumonia* count reached zero after 15 days of storage for all samples treated with spices powder at both levels 0.5% and 1.0%. The difference in *K. pneumonia* count between the control group and all spices powder treated samples was significant (Table 8).

## 4. Discussion

Foodborne pathogens are the foremost cause of illness and death in less developed republics [51]. A meta-analysis study reported that the most prevalent microorganisms isolated from selected African countries were *E. coli*, *Salmonella spp*., *S. aureus*, and *L. monocytogenes* separated from raw and processed foods [51]. In our study, we examined 60 meat samples. A total of 33 isolates have been identified as *S. aureus*, 30 isolates were identified as *E. coli O157: H7*, and 15 isolates were identified as *K. pneumonia.*

*S. aureus* was detected in both fresh and processed meat. However, the highest percentage was in the processed meat samples. In the fresh samples, the highest occurrence was in chicken meat samples. Our results agree with previous studies, as Vorster, et al. [52] reported that *S. aureus* could be detected in 23.4% of all ground beef, 39.5% of all broilers, and 7.1% of all the processed meats bought in Pretoria, South Africa. Furthermore, Shareef, et al. [53] reported that this occurred with 49.99% from all chicken thigh samples present in their study. Adegunloye reported that poultry served as a hazardous source for some pathogens, as it acts as a reservoir for these pathogens, similar to *S. aureus*, that are able to produce enterotoxins [54]. Kitai et al. examined 444 samples of raw chicken meat samples, retailed from 145 different supermarkets in 47 prefectures in Japan, for contamination with *S. aureus* in association with its enterotoxigenicity. *S. aureus* was isolated from 292 (65.8%) of the samples, and from 131 of the 145 supermarkets [55].

The *E. coli O157: H7* is of major concern to the food industries, especially meat and poultry [56]. Our present study results show that. *E. coli O157: H7* could be detected in 3 of 6 fresh veal meat samples, 3 of 6 fresh chicken meat samples, 2 of 3 fresh mutton meat samples, 6 of 12 beef luncheon, 6 of 12 chicken luncheon samples, 4 of 12 ground beef samples, 3 of 6 basterma samples, and 3 of 3 beef burger samples. Another study has shown that *E. coli O157: H7 is* associated with food from animal sources [57]. Stolka, et al. [58] reported that 31% of beef and 7.1% of pork samples were positive for *E. coli O157: H7*. Doyle and Schoeni [57] reported that 3.7% of ground beef, 1.5% of chicken, 2.0% of lamb, and 1.5% of pork samples analyzed in the U.S. were contaminated with *E. coli O157: H7*. The prevalence of *E. coli O157: H7* was higher in the processed meat than fresh meet samples, which was in agreement with a previous study by Cagney, et al. [59].

In our study, *K. pneumonia* was detected in 1 of 6 fresh veal meat samples, 2 of 6 fresh chicken meat samples, 5 of 12 chicken luncheon samples, 1 of 12 ground beef samples, 1 of 6 basterma samples, 1 of 3 beef burger samples, and 4 of 12 beef luncheon samples. However, *K. pneumonia* was not detected in any of fresh mutton meat samples. The highest frequency of *K. pneumonia* was among chicken luncheon samples. Furthermore, in a recent study, they found that the prevalence of *K. pneumonia* was higher in unpacked chicken meat (84.8%), followed by unpacked beef (27.8%) [60].

Essential oils are conventionally used as antifungal and antibacterial agents in natural medicine. The aggregated awareness of modern society and the pharmaceutical industry for medicinal plants makes it vital for the researchers to confirm these properties and discover novel therapeutic agents [61].

The antibacterial activity of essential oils, especially those obtained from leaves, could be related to the presence of two monoterpenes: α pinene and β-pinene. These have been reported to have antimicrobial activity against Gram-positive and Gram-negative bacteria, especially *S. aureus, Bacillus subtilis*, and *E. coli* [62]. The ingredients present in essential oils are synthesized as secondary metabolites by plants to support their survival against environmental stressors, including microbial pathogens [63]. Nowadays, essential oils are employed to cure various medical conditions, such as pain, stress, cancer and infectious disease [63].

In our study, treatment of minced beef samples with essential oils significantly decreased the count of *S. aureus*, *E. coli O157: H7*, and *K. pneumonia* in comparison with control samples during the storage time period. Furthermore, while the microbial count increased in the control samples, the microbial count decreased to reach zero in all treated samples with essential oils, except the *E. coli O157: H7* count in samples treated with marjoram essential oils after 15 days of storage. The decrease in the microbial count could be attributed to the antibacterial activity of these essential oils.

Moreover, the microbial count results for the minced beef samples treated with the spiced powder for the same medicinal plants tested in that study gave almost the same results as essential oils, in which the microbial count reached zero after 15 days of storage in all treated samples with the spices powder, except the *E. coli O157: H7* count in samples treated with marjoram spices powder at 0.5% level only. In light of this result, the decrease in the microbial count could be attributed to the antibacterial activity of these spices powder. Recently, it has been reported that both essential oils and spices powder have antibacterial activity against *Bacillus* species [41].

Abers et al. reported that the cassia, rosemary, and tea essential oils have broad spectrum antimicrobial activity in their airborne evaporative state [63]. Furthermore, they reported that cinnamon, thyme, oregano, frankincense oils, and white fir have moderate broad spectrum antibacterial activity [63].

Moreover, Abers et al. reported that the essential oils in their study proved a variable range of antimicrobial efficacy against microbes. Thus, they suggested that the aerosolized evaporative elements present in the different essential oils have a range of activities on microbial growth [63]. It was previously reported that the crude essential oils have more potent antimicrobial activity as compared to the discrete isolated constituents [64,65].

Eugenol is an aromatic oily liquid extracted from certain essential oils, especially from clove, nutmeg, cinnamon, basil, and bay leaves [66]. A previous study reported that eugenol has antifungal activity with MIC of 9.5 and 8.2 µg/mL against Alternaria alternata and Curvularia lunata, respectively [67]. Eugenol has also been reported to cause impairment of the cell wall and thus lysis of cell in Enterobacter aerogenes [66].

Additionally, a recent study evaluated the antimicrobial effect of six commonly used Brazilian condiments, marjoram, peppermint, basil, rosemary, thyme, and anise against *Clostridium perfringens* strain. The MIC for thyme essential oil was 1.25 mg/mL, 5.0 mg/mL for both basil and marjoram essential oil, and 10 mg/mL for rosemary, anise, and peppermint. With the exception of anise oil which showed only bacteriostatic activity, the rest of condiment essential oils proved both bacteriostatic and bactericidal activity at their respective MICs [68].

The black seeds contain many ingredients in the matrices, such as thymoquinone, dithymoquinone, thymohydroquinone, and thymol [69]. Ugur et al. reported that, concerning the antimicrobial activity of black seed essential oil, the MIC values were 0.5 µg/mL, 2 µg/mL, 64 µg/mL, and 64 µg/mL against *S. aureus* ATCC 29213, *E. faecalis* ATCC 29212, *E. coli* ATCC 25922, and *P. aeruginosa* ATCC 27853 reference strains, respectively [70]. It was also reported that there was antifungal activity as well as antibacterial activity in the same study for the black seed essential oil with an MIC value of 12.5 μg/mL for all the tested microorganisms (*Aspergillus niger, Candida albicans, E. coli, B. subtilis, S. aureus,* and *P. aeruginosa*) [71].

In our study, the results of the microbial count and well-diffusion assay demonstrated that both spices powder and essential oils of cumin, black seeds, clove, cinnamon, and marjoram plants have a broad spectrum antibacterial activity against both Gram-negative and Gram-positive bacteria.

Limitations of the study: One of the limitations of this study is the small sample size examined and the single source of medicinal plants studied. Additionally, we did sequencing for one representative strain from each isolated bacterial group, due to financial restrictions.

## 5. Conclusions

*S. aureus, E. coli O157: H7*, and *K. pneumonia* are associated with food from animal sources, either fresh or processed. The prevalence of them was higher in the processed meat than in fresh meat. The essential oils and spices powder of cumin, black seeds, cloves, cinnamon, and marjoram have an in vitro wide spectrum antibacterial activity. The black seeds (essential oils and spices powder) have the highest antibacterial activity against the tested bacterial strains.

## Figures and Tables

**Table 1:** Frequency of *S. aureus, E. coli O157: H7*, and *K. pneumonia* in tested meat samples.

Food Samples (Number)	Frequency of *S. aureus* (Number, %)	Frequency of *E. coli* O157:H7 (Number, %)	Frequency of *K. pneumonia* (Number, %)
Fresh veal meat (6)	2 (33.3%)	3 (50%)	1 (16.7%)
Fresh chicken meat (6)	3 (50%)	3 (50%)	2 (33.3%)
Fresh mutton meat (3)	00 (0.0%)	2 (66.7%)	0 (0.0%)
Beef luncheon (12)	7 (58.4%)	6 (50%)	4 (33.3%)
Chicken luncheon (12)	7 (58.4%)	6 (50%)	5 (41.7%)
Ground beef (12)	8 (66.7%)	4 (33.4%)	1 (8.4%)
Basterma (6)	3 (50%)	3 (50%)	1 (16.7%)
Beef burger (3)	3 (100%)	3 (100%)	1 (33.3%)
Total (60)	33 (55%)	30 (50%)	15 (25%)

**Table 2:** Sequence analysis of the 16S rDNA gene by BLASTn tool.

Isolates	Name of Closely Associated Strain	Identity %	Gene Bank Accession Number of the Closely Associated Strain
Azhar1	*Staphylococcus aureus subsp. Aureus str. Newbould 305*	100%	EJE55184.1
Azhar2	*Klebsiella pneumoniae* strain *F16KP0070*	100%	CP052585.1
Azhar3	*Escherichia coli strain SCU-176*	99.76%	CP054345.1

**Table 3:** Changes in *S. aureus* count Log (CFU/g) of minced beef treated with essential oils during storage at 4 ± 1 °C for 15 days.

Treatments	Storage Periods (Days)							
	0	3	6	9	12	15	Mean ± S.D	*p* Value
Control	4.339	4.333	4.368	4.415	4.399	4.464	4.386 ± 0.04987	-
Cumin oil 0.25%	4.269	4.049	3.909	3.613	0.00	0.00	2.640 ± 2.056	0.0048
Cumin oil 0.50%	4.218	3.992	3.827	3.545	0.00	0.00	2.597 ± 2.024	0.0028
Black seeds oil 0.25%	4.236	4.359	4.013	3.478	0.00	0.00	2.681 ± 2.099	0.0100
Black seeds oil 0.50%	4.046	3.852	3.177	0.00	0.00	0.00	1.846 ± 2.042	0.0002
Cloves oil 0.25%	4.179	3.978	3.881	3.725	3.323	0.00	3.181 ± 1.585	0.0062
Cloves oil 0.50%	4.159	3.959	3.741	3.398	0.00	0.00	2.543 ± 1.986	0.0016
Cinnamon oil 0.25%	4.189	4.083	3.819	3.279	0.00	0.00	2.562 ± 2.009	0.0023
Cinnamon oil 0.50%	4.284	4.268	3.654	0.00	0.00	0.00	2.034 ± 2.240	0.0022
Marjoram oil 0.25%	4.207	4.121	3.919	3.624	3.042	0.00	3.152 ± 1.601	0.0078
Marjoram oil 0.50%	4.251	3.914	3.663	3.398	0.00	0.00	2.538 ± 1.986	0.0018

*p*: represents the difference between control group and each tested medicinal plant essential oils. S.D = std. deviation.

**Table 4:** Changes in *S. aureus* counts Log (CFU/g) of minced beef treated with spices powder during storage at 4 ± 1 °C for 15 days.

Treatments	Storage Periods (Days)							
	0	3	6	9	12	15	Mean ± S.D	*p* Value
Control	4.358	4.325	4.419	4.454	4.469	4.493	4.420 ± 0.06596	-
Cumin 0.50%	4.284	4.291	3.786	3.492	3.042	0.00	3.149 ± 1.615	0.0086
Cumin 1.0%	4.233	4.046	3.887	3.613	0.00	0.00	2.630 ± 2.047	0.0033
Black seeds 0.50%	4.188	4.018	3.839	0.00	0.00	0.00	2.008 ± 2.202	0.0010
Black seeds 1.0%	4.179	3.992	3.741	0.00	0.00	0.00	1.985 ± 2.179	0.0007
Cloves 0.50%	4.182	4.049	3.833	3.613	0.00	0.00	2.613 ± 2.033	0.0023
Cloves 1.0%	4.124	3.978	3.691	3.079	0.00	0.00	2.479 ± 1.953	0.0008
Cinnamon 0.50%	4.188	4.124	3.858	3.673	3.492	0.00	3.223 ± 1.601	0.0074
Cinnamon 1.0%	4.144	4.104	3.756	0.00	0.00	0.00	2.001 ± 2.196	0.0008
Marjoram 0.50%	4.218	4.124	3.978	3.699	2.699	0.00	3.120 ± 1.625	0.0083
Marjoram 1.0%	4.144	4.076	3.864	3.613	3.463	0.00	3.193 ± 1.586	0.0047

*p*: represents the difference between control group and each tested medicinal plant spices powder. S.D = std. deviation.

**Table 5:** Changes in *E. coli O157: H7* count Log (CFU/g) of minced beef treated with essential oils during storage at 4 ± 1 °C for 15 days.

Treatments	Storage Periods (Days)							
	0	3	6	9	12	15	Mean ± S.D	*p* Value
Control	4.325	4.308	4.345	4.373	4.405	4.461	4.370 ± 0.0565	-
Cumin oil 0.25%	4.277	4.177	4.049	3.949	3.492	0.00	3.324 ± 1.651	0.0111
Cumin oil 0.5%	4.265	4.159	4.083	3.839	3.343	0.00	3.282 ± 1.641	0.0078
Black seeds oil 0.25%	4.239	4.065	3.959	3.858	3.114	0.00	3.206 ± 1.618	0.0029
Black seeds oil 0.5%	4.209	3.949	3.733	3.079	0.00	0.00	2.495 ± 1.969	0.0003
Cloves oil 0.25%	4.241	4.072	3.929	3.623	0.00	0.00	2.644 ± 2.058	0.0013
Cloves oil 0.5%	4.162	4.118	3.978	3.506	0.00	0.00	2.627 ± 2.048	0.0015
Cinnamon oil 0.25%	4.228	4.156	4.46	3.959	3.624	0.00	3.405 ± 1.691	0.0272
Cinnamon oil 0.5%	4.046	4.083	3.869	2.955	2.612	0.00	2.928 ± 1.558	0.0004
Marjoram oil 0.25%	4.223	4.089	4.005	3.945	3.869	3.279	3.902 ± 0.3285	0.0132
Marjoram oil 0.5%	4.258	4.061	3.987	3.89	3.771	3.415	3.897 ± 0.2876	0.0104

*p*: represents the difference between control group and each tested medicinal plant essential oils. S.D = std. deviation.

**Table 6:** Changes in *E. coli O157:H7* counts Log (CFU/g) of minced beef treated with spices powder during storage at 4 ± 1 °C up to 15 days.

Treatments	Storage Periods (Days)							
	0	3	6	9	12	15	Mean ± S.D	*p* Value
Control	4.349	4.359	4.377	4.417	4.405	4.455	4.394 ± 0.03977	-
Cumin 0.5%	4.282	4.228	4.083	3.964	0.00	0.00	2.760 ± 2.140	0.0068
Cumin 1.0%	4.277	4.185	3.323	2.584	1.688	0.00	2.676 ± 1.637	0.0014
Black seeds 0.5%	4.258	4.111	4.057	3.876	0.00	0.00	2.717 ± 2.108	0.0031
Black seeds 1.0%	4.236	3.983	3.799	3.323	0.00	0.00	2.557 ± 2.003	0.0004
Cloves 0.5%	4.261	4.087	3.959	3.699	0.00	0.00	2.668 ± 2.074	0.0016
Cloves 1.0%	4.191	4.124	4.005	3.557	0.00	0.00	2.646 ± 2.062	0.0017
Cinnamon 0.5%	4.083	4.101	3.909	3.343	3.079	0.00	3.086 ± 1.568	0.0011
Cinnamon 1.0%	4.239	4.162	4.083	4.026	0.00	0.00	2.752 ± 2.133	0.0056
Marjoram 0.5%	4.233	4.111	4.046	3.987	3.819	3.323	3.920 ± 0.3229	0.0140
Marjoram 1.0%	4.275	4.079	4.038	3.909	3.725	0.00	3.338 ± 1.645	0.0055

*p*: represents the difference between control group and each tested medicinal plant spices powder.

**Table 7:** Changes in *K. pneumonia* count Log (CFU/g) of minced beef treated with essential oils during storage at 4 ± 1 °C for 15 days.

Treatments	Storage Periods (Days)							
	0	3	6	9	12	15	Mean ± S.D	*p* Value
Control	4.297	4.347	4.364	4.435	4.452	4.479	4.396 ± 0.07039	-
Cumin oil 0.25%	4.239	3.992	3.807	3.463	0.00	0.00	2.584 ± 2.017	0.0036
Cumin oil 0.5%	4.188	3.939	3.613	0.00	0.00	0.00	1.957 ± 2.151	0.0010
Black seeds oil 0.25%	4.221	4.076	3.929	3.579	0.00	0.00	2.634 ± 2.052	0.0050
Black seeds oil 0.5%	4.076	3.819	0.00	0.00	0.00	0.00	1.316 ± 2.040	0.0002
Cloves oil 0.25%	4.185	4.087	3.813	3.613	0.00	0.00	2.616 ± 2.037	0.0047
Cloves oil 0.5%	4.177	3.644	0.00	0.00	0.00	0.00	1.304 ± 2.026	0.0002
Cinnamon oil 0.25%	4.228	4.153	4.079	3.749	3.398	0.00	3.268 ± 1.630	0.0212
Cinnamon oil 0.5%	4.149	3.929	3.624	0.00	0.00	0.00	1.950 ± 2.143	0.0008
Marjoram oil 0.25%	4.291	4.244	3.978	3.708	3.519	0.00	3.290 ± 1.639	0.0281
Marjoram oil 0.5%	4.144	3.869	3.557	3.177	0.00	0.00	2.458 ± 1.931	0.0011

*p*: represents the difference between control group and each tested medicinal plant essential oils. S.D = std. deviation.

**Table 8:** Changes in *K. pneumonia* counts Log (CFU/g) of minced beef treated with spices powder during storage at 4 ± 1 °C for 15 days.

Treatments	Storage Periods (Days)							
	0	3	6	9	12	15	Mean ± S.D	*p* Value
Control	4.299	4.343	4.358	4.409	4.437	4.455	4.384 ± 0.06009	-
Cumin 0.5%	4.246	4.009	3.852	3.519	0.00	0.00	2.604 ± 2.031	0.0027
Cumin 1.0%	4.207	3.969	3.741	3.415	0.00	0.00	2.555 ± 1.997	0.0017
Black seeds 0.5%	4.231	4.345	3.959	3.644	0.00	0.00	2.697 ± 2.103	0.0088
Black seeds 1.0%	4.087	3.852	3.302	0.00	0.00	0.00	1.874 ± 2.068	0.0002
Cloves 0.5%	4.207	4.114	3.864	3.741	0.00	0.00	2.654 ± 2.063	0.0044
Cloves 1.0%	4.202	3.749	3.569	0.00	0.00	0.00	1.920 ± 2.113	0.0003
Cinnamon 0.5%	4.248	4.194	4.118	3.852	3.592	0.00	3.334 ± 1.652	0.0233
Cinnamon 1.0%	4.177	3.964	3.733	3.177	0.00	0.00	2.509 ± 1.971	0.0011
Marjoram 0.5%	4.297	4.262	4.038	3.819	3.624	0.00	3.340 ± 1.656	0.0284
Marjoram 1.0%	4.156	3.909	3.654	3.398	3.042	0.00	3.027 ± 1.533	0.0016

*p*: represents the difference between control group and each tested medicinal plant spices powder. S.D = std. deviation.
